# From Work Well-Being to Burnout: A Hypothetical Phase Model

**DOI:** 10.3389/fnins.2020.00360

**Published:** 2020-04-30

**Authors:** L. P. Morera, J. I. Gallea, M. A. Trógolo, M. E. Guido, L. A. Medrano

**Affiliations:** ^1^Instituto de Organizaciones Saludables, Universidad Siglo 21, Córdoba, Argentina; ^2^Departamento de Biología Química, Universidad Nacional de Córdoba, Córdoba, Argentina; ^3^Pontifica Universidad Católica Madre y Maestra, Vicerrectoría de Investigación, Santiago de los Caballeros, Dominican Republic

**Keywords:** burnout, well-being, cortisol, stress, HPA, workplace, engagement

## Abstract

Upon exposure to chronic stressors, how do individuals move from being in a healthy state to a burnout? Strikingly in literature, this has prevailed a categorical view rather than a dimensional one, thus the underlying process that explains the transition from one state to another remains unclear. The aims of the present study are (a) to examine intermediate states between work engagement and burnout using cluster analysis and (b) to examine cortisol differences across these states. Two-hundred and eighty-one Argentine workers completed self-report measures of work engagement and burnout. Salivary cortisol was measured at three time-points: immediately after awakening and 30 and 40min thereafter. Results showed four different states based on the scores in cynicism, exhaustion, vigor, and dedication: engaged, strained, cynical, and burned-out. Cortisol levels were found to be moderate in the engaged state, increased in the strained and cynical states, and decreased in the burned-out state. The increase/decrease in cortisol across the four stages reconciles apparent contradictory findings regarding hypercortisolism and hypocortisolism, and suggests that they may represent different phases in the transition from engagement to burnout. A phase model from engagement to burnout is proposed and future research aimed at evaluating this model is suggested.

## Introduction

Workers account for half of the world’s population and represent the largest contributors to economic and social development (World Health Organization, 2007). Over the last decades, globalization, privatization, and liberalization has caused significant changes at work, such as increasing demands, the need to adopt new types of works, higher pressure of productivity and quality of work, and time pressure (Spontón et al., 2019). As a result, work-related stress phenomena such as burnout have increased rapidly, representing a main risk factor for health in Western societies (Leka et al., 2010).

Burnout is a work-related syndrome that emerges in response to chronic stress (Maslach et al., 2001). Empirical research has demonstrated the negative influence of burnout on individuals’ psychological and physical health, as well as on an organization’s effectiveness (Carod Artal and Vázquez-Cabrera, 2013). There are a number of factors that contribute to burnout development, including behavior, lifetime experiences, environment, and neurophysiology (for a review, see McEwen and Stellar, 1993; Leiter and Maslach, 2017); however, the pathway connecting stressful life events and burnout are still a matter of discussion and the exact physiological mechanisms involved remain elusive.

Past research has repeatedly shown cortisol to be associated with stress and it has hence been proposed as a biomarker of burnout. Despite the general consensus around this hormone and its role in mediating responses to stress, findings on the burnout-cortisol relationship have been inconsistent. For instance, high levels of cortisol have been positively associated with burnout (Penz et al., 2018), whereas other studies have found a negative relationship between these variables (Oosterholt et al., 2015; Pilger et al., 2018). Further research is therefore needed to gain a better understanding of the physiological mechanisms underlying burnout.

The focus on burnout has largely neglected research on positive experiences in the workplace, until recently. The shift to the positive side of people’s work experience was partly due to the increased recognition that reducing stress or discomfort is not equivalent to increasing well-being and health (Salanova et al., 2016). As a consequence, studies on work engagement, a positive work-related state of well-being, have grown exponentially over the last years (Bakker et al., 2011).

Work engagement has been linked with several positive outcomes, including organizational commitment, extra-role behavior, personal initiative, organizational performance, and quality of service (Schaufeli and Salanova, 2010). In addition, some studies found a relationship between cortisol and work engagement (Langelaan et al., 2006; Bakker et al., 2011); however, the extent to which glucocorticoids mediate the necessary physiological activation of work engagement remains unclear.

Work engagement and burnout are conceptualized as each other’s opposite (Maslach and Leiter, 1997), most of the research tapping the two simultaneously has been conducted from a categorical rather than a dimensional viewpoint (Holmes and Patrick, 2018). This approach entails certain limitations since it does not take into account intermediate states or phases between work engagement and burnout. There has been a steady increase in the number of publications examining relations between engagement, burnout and cortisol (Langelaan et al., 2006; Ortiz Valdés and Vega-Michel, 2009; Fernández-Sánchez et al., 2017), although most of the published literature is essentially descriptive. To the best of our knowledge, a model explaining the progression from work engagement to burnout is currently lacking.

Investigating the progression between engagement and burnout is in line with current efforts to improve the classification system of psychiatry and psychopathology. Over the past several years, a competing vision, namely, the Research Domain Criteria (RDoC) initiative launched by the National Institute of Mental Health, has emerged in response to accumulating anomalies within the Diagnostic and Statistical Manual of Mental Disorders (DSM) and the International Statistical Classification of Diseases and Related Health Problems (ICD) system (Lilienfeld and Treadway, 2016). The DSM-ICD model assumes that disorders are discrete entities, which differ qualitatively from normality and from each other. In contrast, RDoC was developed in large part to support research into the etiologies of mental disorder. Its ambitious goal is to understand how functional deviations in various brain and behavioral response system interact to result in mental disorder, while emphasizing that these processes are progressive rather than static. The approach is fully and explicitly dimensional, not simply across the severity range of a diagnosed disorder but across the entire span of normal to abnormal functioning (Clark et al., 2017). In this framework, research is needed to propose hypotheses (and not just test them) on the progression between well-being and mental disorders.

The present study builds on previous research and contributes to the literature in at least two ways. Firstly, we use cluster analysis to examine different patterns of combinations of exhaustion, cynicism, vigor, and dedication and postulate intermediate phases between work engagement and burnout. This method has been proposed not only as a powerful tool for detecting structure in data sets, but also for identifying the features underlying the progression from a healthy to an unhealthy state (Ahlqvist et al., 2018; Alashwal et al., 2019). Furthermore, identifying patient subpopulations under the hypothesis that patients with similar conditions are likely to share a common disease mechanism, represents a cornerstone for precision medicine (Robinson, 2012; Saria and Goldenberg, 2015; Gligorijević et al., 2016; Feher et al., 2018). Secondly, we explore hypothalamic pituitary adrenal axis (HPA) and cortisol involvement and activation throughout the intermediate phases. By doing so, we provide new insights that contribute to integrate inconsistent research findings observed in the literature regarding burnout, engagement and cortisol.

### Background

#### Burnout and Cortisol

Glucocorticoids and HPA axis involvement have been extensively described under acute stress, but their functioning throughout chronic stress and particularly in the workplace still requires clarification (Elbau et al., 2018; Rohleder, 2018; Sunwoo et al., 2019).

Burnout has been defined as a work-related syndrome that emerges in response to chronic stress, and is characterized by the presence of (a) feelings of energy depletion or emotional and physical exhaustion and (b) increased mental distance from one’s job, or feelings of negativism or cynicism related to one’s job (Halbesleben and Demerouti, 2005).

Some studies indicate that in burnout, the HPA axis is under chronic and persistent activation (Adam et al., 2006; Rohleder, 2018). According to these studies, upon stressor exposure the HPA axis releases glucocorticoids. This result in the mobilization of energetic resources, including the stimulation of gluconeogenesis, with subsequently increased levels of circulating glucose and the down-ward regulation of inflammatory processes, facilitating individual to cope with stressful situations. However, the persistent activation of the HPA axis requires high energy consumption, and failure to restore balance causes wear, ultimately leading to exhaustion.

Cynicism has been conceptualized as defensive coping resulting from exhaustion, involving emotional (negative affect) and cognitive-motivational components (Volpe et al., 2014; Maslach, 2015). Under chronic stress, it has been shown that increased levels of glucocorticoids and an uncontrollable perception of the stressor may lead to behaviors related to cynicism, such as withdrawal and disengagement (Miller et al., 2007). Furthermore, this particular outcome has been related to the disturbance of the dopaminergic and serotonergic neurobiological pathways (Tafet et al., 2001; Seo et al., 2008; Panksepp, 2010; Tafet and Nemeroff, 2016).

#### Engagement and Cortisol

Engagement can be understood as a positive work-related affective-cognitive state of mind characterized by vigor and dedication. Rather than a specific, momentary state, engagement refers to a more persistent state that is not limited to a particular object, event or situation (Schaufeli et al., 2002). Vigor refers to high levels of energy and mental resistance while working and the desire to invest effort in the work being carried out even in the face of difficulties. Dedication implies high work involvement along with feelings of meaning, enthusiasm, inspiration, pride, and being challenged by work (Schaufeli, 2004).

Although engagement represents a healthy and positive state of well-being (Feldon et al., 2019), engaged employees need to mobilize resources to face job demands, giving rise to a state of activation-tension. Glucocorticoids, among other mediators, participate in an orchestrated manner in this state. Cortisol is thus involved in pathways that mediate key processes necessary to achieve and maintain engagement, reinforce both rewarding stimuli and the subjective experience of pleasure and cope with demands.

## Materials and Methods

### Participants

A self-selected sample of 281 workers (41% female and 59% male) from Córdoba, Argentina, participated in the study. The sample comprised workers of both sex (41% female and 59% male) aged between 20 and 60years (*M*=36.44; SD=8.28). 44% of the participants worked in the public sector (work start time 9 a.m.) and 56% in private companies (work start time 8 a.m.).

Participants were asked to collect three saliva samples in the morning on a workday: immediately after awakening, 15 and 45min thereafter. Subjects were asked to refrain from drinking, eating, and brushing their teeth before the collection of all three saliva samples. Inclusion criteria for the study were, possession of appropriate language (able to read and complete questionnaires in Spanish), of both sexes, having signed the corresponding informed consent and being active workers with at least 2 months of seniority in the institution at the time of the sampling. All participants were asked to provide information regarding potential covariates that could affect cortisol levels and therefore to be considered as exclusion criteria, they should have had the use of systemic or topical steroids in the last 4 weeks, intense exercise prior to sampling, report of consumption of steroid-based anti-inflammatory drugs, oral injuries or diseases, alcoholism, chemotherapy, prolonged corticotherapy, autoimmune diseases, and infection.

### Measures

#### Burnout and Work Engagement

Exhaustion and cynicism were assessed with the Argentinean version (Spontón et al., 2019) of the Maslach Burnout Inventory-General Survey (MB-GS; Schaufeli and Buunk, 1996). The exhaustion scale comprised five items (e.g., “I feel emotionally drained from my work”) and the cynicism scale included four items (e.g., “I have become less enthusiastic about my work”). Vigor and dedication were assessed with the Argentinean version (Spontón et al., 2012) of the Utrecht Work Engagement Scale (UWES; Schaufeli et al., 2002). The vigor scale included six items (e.g., “at my work, I feel bursting with energy”) and the dedication scale also included six items (e.g., “I am enthusiastic about my work”). All items tapping core burnout and work engagement dimensions were rated on a 7-point frequency scale, ranging from 0 (*never*) to 6 (*daily*). In order to avoid response bias, we randomly merged all burnout and work engagement items into one questionnaire.

#### Salivary Cortisol

Salivary cortisol was collected at three time-points to determine the cortisol awakening response (CAR): immediately after awakening and 30 and 45min thereafter (Powell and Schlotz, 2012). All samples were then stored at 4°C until sent to the laboratory. Once in the laboratory, saliva samples were centrifuged for 5-min at 2000 rpm to extract saliva with low viscosity, and subsequently transferred to 1.5 mL tubes and stored in a freezer at −80°C until analysis. After thawing, 20 μl of salivary samples were transferred to a sample cup and cortisol was estimated using a cortisol RP Elecsys Kit (Roche Diagnostics, United States) in a cobas e 411 analyzer. Summary indexes of CAR included the area under the curve with respect to ground (AUCg) and the area under the curve with respect to increase (AUCi) (Pruessner et al., 2003).

#### Socio-Demographic Questionnaire

Personal details were obtained in relation to sex, age, and type of sector in which participants worked.

### Procedure

Employees were contacted via e-mail and invited to participate in a study of well-being at work. The response rate was high (86%). Those who agreed to participate signed a written consent and responded a paper-and-pencil questionnaire with all the scales and an accompanying letter explaining the objectives of the study. Then, participants were provided with three plastic tubes (Corning LS tubes of 15 mL) and detailed instructions on collecting saliva, emphasizing the need to strictly follow the time schedule and refrain from drinking, eating, and brushing their teeth before collecting each of the three saliva samples (Stalder et al., 2016). Data were collected between April and June 2018. This study was approved by the Ethics Committee of the Faculty of Medical Sciences, National University of Córdoba. The participants in this study did not receive any kind of compensation for participating and all of them gave written prior informed consent. Finally, the study was performed following the Helsinki Declaration for medical studies in humans.

### Data Analysis

All statistical analyses were performed using SPSS 23.0. Firstly, internal consistencies (Cronbach’s alpha), descriptive statistics and correlations among variables were computed. Secondly, we explored different intermediate states within the engagement-burnout continuum by means of non-hierarchical *k*-means cluster analysis. This technique enables the grouping of individuals based on the similarity of their responses to a set of variables by way of an iterative process (Clatworthy et al., 2005). In the present study, employees’ scores on exhaustion, cynicism, vigor, and dedication were used as input for the analysis. Since we had no prior expectations on the number of intermediate states, different cluster solutions were examined and subsequently compared in order to determine the most appropriate solution in terms of parsimony and interpretability. Finally, differences in cortisol levels (awakening, +30, +45, AUCg, and AUCi) across phases identified were examined. Since AUCi values can be positive and negative and we are interested only in comparing the intensity of changes in cortisol, each value was square transformed to make them all positive (Harrison et al., 2009). Typically, comparison between more than two group phases in the present study and is done using analysis of variance (ANOVA). However, this procedure often requires fairly large sample sizes to achieve adequate statistical power (Olejnik and Algina, 2003). Since some of the groups were small, between-group differences were calculated using Student’s *t* test with Bonferroni adjustment to control for Type I error associated with pairwise multiple comparisons (Jaccard and Sheng, 1984). Hedge’s *g* was computed to determine the effect size of mean differences; this is a more appropriate tool when dealing with different group sizes (Lakens, 2013) as in the present study, since it provides a measure of effect size weighted according to the relative size of each group.

## Results

### Preliminary Analysis

Table 1 displays the means, standard deviations, skewness, internal consistencies (Cronbach’s alpha) and bivariate correlations among the study variables. As seen in the table, all the variables were slightly skewed and yielded acceptable alpha values (≥0.70; Nunnally, 1975). Correlations among the variables were significant, ranging from −0.68 to 0.41, indicating that although related they are non-redundant (i.e., any correlation ≥0.90 is suggestive of redundancy; Tabachnick and Fidell, 2007) and multicollinearity among variables was therefore discarded.

**TABLE 1:** Descriptive statistics, internal consistency and correlations among the study variables.

	Mean	SD	Skewness	A	B	C	D
A. Exhaustion	13.61	5.64	0.24	0.74			
B. Cynicism	7.42	4.92	1.12	0.47**	0.77		
C. Vigor	16.28	3.53	–0.77	–0.20**	–0.43**	0.77	
D. Dedication	14.56	4.72	–0.54	–0.28**	–0.68**	0.45**	0.88

Cronbach’s alpha values are on the diagonal. ** *p*<0.01 (two-tailed).

### Cluster Analysis

**FIGURE 1:**
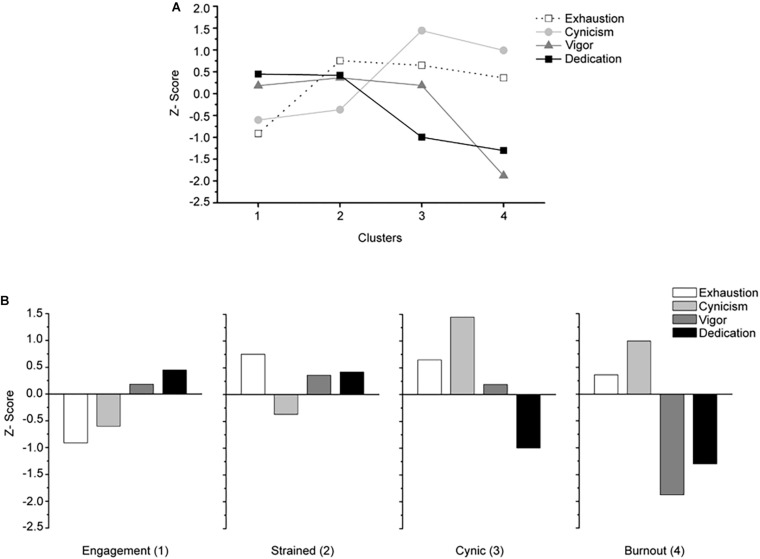
**(A)** Progression of core dimensions of burnout (exhaustion and cynicism) and engagement (vigor and dedication) throughout clusters. **(B)** Participant’s standardized mean scores on the engagement and burnout dimensions as a function of group membership.

Three to six cluster solutions were examined, of which the four-solution one proved to be the more parsimonious and interpretable. Figure 1 presents standardized mean scores on the clustering variables by group. Analogous to Cohen’s *d*, an SD of 0.2 is considered a small effect, 0.5 a medium effect and 0.8 a large effect (Cohen, 1988). Cluster 1 (*n*=122, 42.1%) is characterized by employees with the lowest scores in exhaustion (*Z*=–0.90) and cynicism (*Z*=–0.57) and the highest scores in vigor (*Z*=0.25) and dedication (*Z*=0.48), therefore representing the *engaged* group. Cluster 2 (*n*=84, 29.1%) is characterized by employees who display similar levels of cynicism (*Z*=−0.41), vigor (*Z*=0.34), and dedication (*Z*=0.46) to cluster 1. However, they also had notably higher levels of exhaustion (*Z*=0.82). Accordingly, we labeled this group as the *strained* group. Cluster 3 (*n*=49, 17%) is characterized by employees with the highest level of cynicism (*Z*=1.44), moderate exhaustion (*Z*=0.64), slightly above-average scores on vigor (*Z*=0.18), and low scores on dedication (*Z*=–0.99), thus representing the *cynical* group. Cluster 4 (*n*=34, 11.8%) is characterized by employees with the lowest levels of dedication (*Z*=–1.30) and vigor (*Z* = −1.87), moderate levels of exhaustion (*Z*=0.40) and higher levels of cynicism (*Z*=0.99), and represents the *burned-out* group.

### Cortisol

Differences in cortisol among groups are presented in Table 2. The engaged group showed significantly lower cortisol levels at +30min (Hedge’s *g*=0.37) and lower AUCg values (*g*=0.38) than the strained group. Engaged workers also exhibited significantly lower cortisol levels immediately after awakening (*g*=*0.48*), at +30min (*g*=0.56), and lower AUCg (*g*=0.53) and AUCi (*g*=0.39) values than the cynical group. The cynical group showed significantly higher cortisol at +45min (*g*=0.55) than the burned-out group. There were non-significant differences in cortisol between the burned-out and engaged groups, and between the strained and cynical groups.

**TABLE 2:** Cortisol differences among subgroups of workers.

	Engaged (1)	Strained (2)	Cynical (3)	Burned-out (4)	Significant*
	Mean (SD)	Mean (SD)	Mean (SD)	Mean (SD)	
*CORT-T0*	0.66 (0.34)	0.75 (0.39)	0.84 (0.45)	0.78 (0.35)	1 < 3
*CORT-T30*	0.81 (0.42)	0.99 (0.56)	1.05 (0.44)	0.84 (0.40)	1 < 3, 1 < 2
*CORT-T45*	0.75 (0.51)	0.85 (0.41)	0.89 (0.42)	0.68 (0.31)	3 > 4
*AUCg*	32.88 (14.57)	38.72 (15.75)	41.36 (18.53)	34.85 (15.90)	1 < 3, 1 < 2
*AUCi*^2^	168.71 (368.71)	182.31 (341.17)	323.89 (474.63)	138.92 (267.85)	1 < 3

* *p*<*0.05* (with Bonferroni correction: 0.05/20=0.0025).

## Discussion

A large body of research data supports the idea that most symptoms associated with mental disorders exist on spectrum, with many of them present to some degree even in the “normal” population (Clark et al., 2017). To better understand mental disorders, new hypotheses about the progression between well-being and mental disorders need to be proposed.

Engagement and burnout have been conceived as polar opposites of occupational well-being. As stated by Maslach and Leiter (1997), individuals may typically feel bursting with high energy (vigor) and dedication when starting a new job, but under stressful conditions, these states of energy and dedication may erode and convert into low energy (exhaustion) and low dedication (cynicism). However, surprisingly little effort has been dedicated to develop a model that attempts to explain the progression from engagement to burnout, looking at both psychological and physiological measurements. In the present study we address this issue by using cluster analysis to identifying the phases that may underlie the trajectory from engagement to burnout.

**FIGURE 2:**
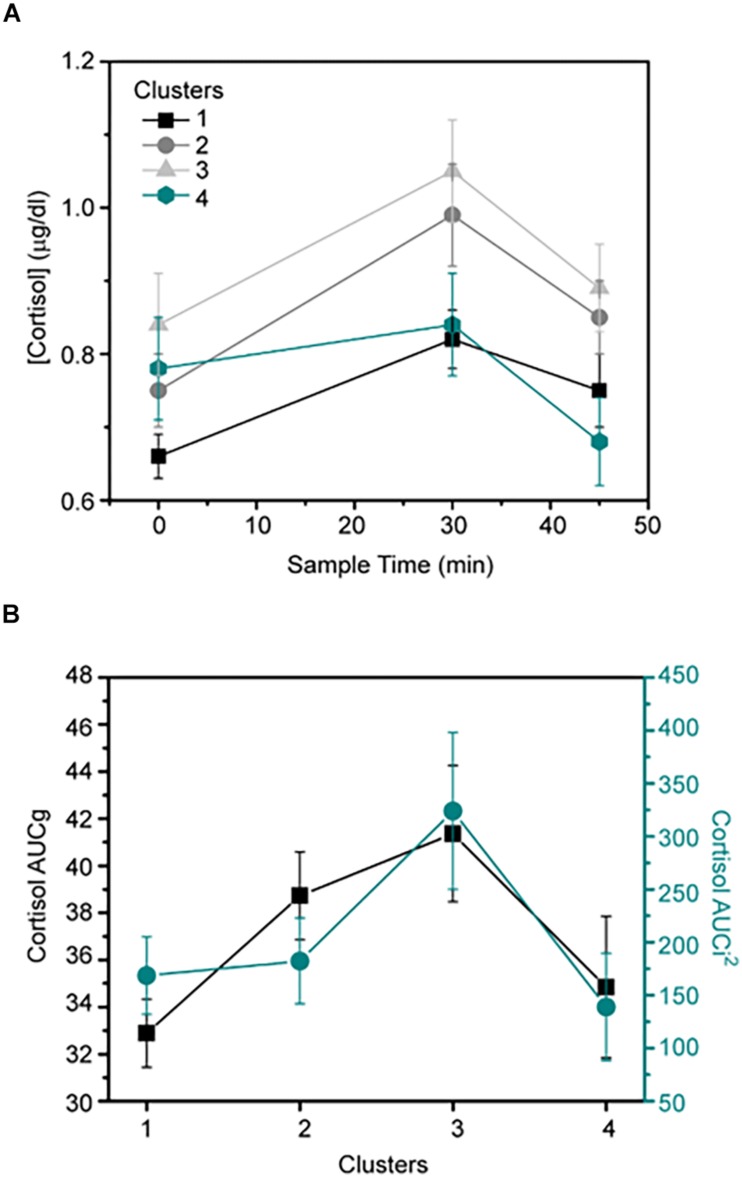
**(A)** Comparisons of cortisol awakening response (CAR) among four clusters, cluster 1 (engaged), cluster 2 (strained), cluster 3 (cynical), and cluster 4 (burned-out). **(B)** AUCg and AUCi^2^ levels throughout the four-cluster solution.

**FIGURE 3:**
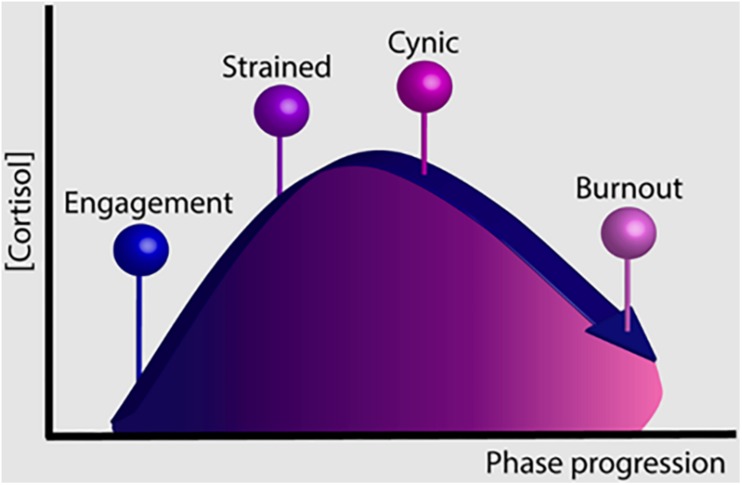
Hypothetical phase model on the trajectory from engagement to burnout.

Four different groups emerged from our findings: engaged, strained, cynical, and burned-out. The engaged group displayed high vigor and dedication, and low exhaustion and cynicism; the burned-out group displayed the opposite pattern of scores in dedication, cynicism, exhaustion, and vigor. The strained and cynical groups revealed scores that were generally “in between” the scores of the engaged group on the one hand and the burned-out group on the other. The only exception was cynicism, which was highest in the cynical group. These patterns of results suggest that strained and cynicism may represent intermediate states between engagement and burnout. In addition, differences in cortisol and the HPA axis functioning across the groups suggest that there may be a temporal sequence between engagement, strained, cynicism, and burnout (Figure 3). In particular, AUCg levels increased in the strained and cynical groups, and declined in the burned-out group (Figure 2A). These results are consistent with meta-analytic studies suggesting that chronic stress both increases and decreases HPA axis activity, but do so at different times over the course of the threat (Miller et al., 2007).

In the *engagement* phase, AUCi levels indicate a moderate increase in cortisol levels upon awakening. These results are consistent with previous studies linking a moderate increase in cortisol level upon awakening with a healthy HPA axis response (Kalafatakis et al., 2018). Contrary to the prevailing view of cortisol as “the bad guy” (McEwen, 2019), our findings also suggest that the moderate release of cortisol may fuel energic states such as vigor and that this particular neurohormonal response mediates a cognitive engaged state (Welch, 2013). Moreover, observed cortisol levels could be associated with successful coping with stressful events (Powell and Schlotz, 2012).

The second phase, *strained*, is characterized by high levels of dedication and vigor, but also high exhaustion and increased levels of cortisol as reflected by AUCg values. Presumably, workers in this phase are exposed to increasing job demands (Demerouti et al., 2001) but which they still perceive as controllable. Stressors or demands perceived as manageable may activate the HPA axis, increasing the release of cortisol to provide the metabolic support for active coping efforts (Miller et al., 2007). Cortisol may play a buffering role such that once a certain level of glucocorticoids is surpassed and sustained over time, it unfolds an additional imbalance in other neurobiological pathways (Tafet and Nemeroff, 2016). It has also been hypothesized that exhaustion and increased cortisol levels might leave individuals more vulnerable to cortisol effects (Penz et al., 2018).

The third phase, *cynicism*, is characterized by the highest levels in cynicism, a marked dropdown in dedication and the highest AUCi and AUCg levels (Figure 2A). Unlike the strained phase, job demands here have likely become uncontrollable, resulting in diminished HPA activity (Miller et al., 2007). This overall higher level of cortisol and increased morning secretion have been systematically associated with mental illness (Owens et al., 2014). It has been pointed out that higher and sustained glucocorticoid concentrations exert negative effects on the serotoninergic system. Glucocorticoids increase 5-hydroxytryptamine (5-HT) uptake, mediated by an increased expression of the 5-HTT gene (Tafet et al., 2001). A diminished concentration and limited availability of serotonin in the synaptic cleft would limit its effects in both pre- and post-synaptic receptors. Furthermore, environmental stressors may provoke increased concentrations of dopamine in the mesocortical pathway, exerting an exaggerated activation in the face of moderately negative stimuli. Higher and sustained levels of cortisol could therefore explain the apparition of the strained phase in the first place, which would then give rise to the cynicism phase.

The fourth phase, *burnout*, is characterized by the lowest level of vigor and dedication, moderate exhaustion, and high cynicism but somewhat lower than that in the cynicism phase. It seems likely that as the burnout symptomatology advances, GC does the same, but once a certain level of severity is reached in the upper range of burnout symptoms, HPA axis activity starts to decrease, displaying the transition from hyper- to hypocortisolism (see Figure 2B). Our results are supported by the conclusions reached in the seminal work by Miller et al. (2007): “…with stress that is more severe and persists longer, uncontrollability is thought to result in diminished HPA activity. This blunting may underlie the withdrawal and disengagement behaviors that often accompany uncontrollable chronic stress.” Finally, recent published work from Penz et al. (2018) partially supports our results, they hypothesized about a two-stage process in HPA axis activation in situations of prolonged exposure to stressors.

### Limitations and Future Research

There are certain limitations of the study that should be acknowledged. In particular, our proposed phase model posits that cynical and strained groups may represent intermediate phases in the trajectory from engagement to burnout. Since our study was cross-sectional, it is critical that future research longitudinally examine the intra-individual developmental paths from engagement to burnout. The use of longitudinal within-subject design and latent profile analysis would be particularly useful not only to empirically test the proposed phase model, but also to examine the patterns of stability and change over time and the factors that mediate the transition from one phase to another. For example, as mentioned previously, it has been suggested that under chronic stressors, perceived uncontrollability results in diminished HPA activity and that this decrease may lie behind the withdrawal behaviors that correspond to cynicism (Heim et al., 2000). As self-efficacy moderates the influence of job stressors on perceived controllability (Liu et al., 2018), one might argue that employees with a low sense of self-efficacy facing high job demands are more likely to perceive these demands as uncontrollable, resulting in lowering of the cortisol secretion required to cope with demands and distancing from work as a defensive strategy, transitioning into the cynicism phase. In contrast, employees exposed to high job demands but with a high sense of self-efficacy are more likely to perceive these as manageable, increasing mobilization of energetic resources – high HPA activity – to actively cope with demands, resulting in more exhaustion due to increased effort, but not in withdrawal. Likewise, some studies showed that psychological detachment from work moderate the impact of job demands on exhaustion, such that employees with high demands and low psychological detachment exhibit increased exhaustion and decreased engagement, whereas employees with high job demands but high psychological detachment maintain high levels of engagement over time (Sonnentag et al., 2010). In addition, there is evidence that low psychological detachment is associated with increased cortisol secretion (Cropley et al., 2015). Collectively, these results suggest that psychological detachment from work may buffer the influence of job demands, keeping employees engaged even when job demands are high and, conversely, low psychological detachment may prolong stress-related physiological activation resulting in exhaustion. Addressing the individual and social factors underlying the shift from one phase to another will be of paramount importance for developing early interventions to prevent the progression toward burnout.

## Conclusion

Literature on the relationship between work engagement and burnout is largely descriptive. To date, no study has attempted to explain how employees transit from one state to another, particularly from a physiological approach. In this study, based on cluster analysis and examination of cortisol and HPA activity across the clusters identified, we propose a model with different phases involved in the transition from engagement to burnout. A longitudinal assessment of the proposed model along with cortisol measurement would be useful for confirming the present findings.

## Data Availability Statement

The datasets generated for this study are available on request to the corresponding author.

## Ethics Statement

The studies involving human participants were reviewed and approved by the Ethics Committee of the Faculty of Medical Sciences, National University of Córdoba. The patients/participants provided their written informed consent to participate in this study.

## Author Contributions

LPM and LAM conceived the study. JG and LPM participated in study design and acquisition of the data. MT made substantial contributions to the study design, and data analysis, MG critically revised the manuscript. All authors made substantial contributions to interpretation of the data, to drafting the manuscript or revising it critically for important intellectual content.

## Conflict of Interest

The authors declare that the research was conducted in the absence of any commercial or financial relationships that could be construed as a potential conflict of interest.
